# Seed Disinfestation Practices to Control Seed-Borne Fungi and Bacteria in Home Production of Sprouts

**DOI:** 10.3390/foods12040747

**Published:** 2023-02-08

**Authors:** Gregory S. Gilbert, Alyssa Diaz, Haylee A. Bregoff

**Affiliations:** Environmental Studies Department, University of California, Santa Cruz, CA 95064, USA

**Keywords:** seed microbiology, bacteria, fungi, seed disinfection, seed sterilization, seed sprouts, food safety, hypochlorous acid, sodium hypochlorite, home sprout production

## Abstract

Concern over microbial contamination limits the adoption of home production of sprouts as a nutritious and sustainable food. Simple, accessible approaches to seed disinfection could support safe home seed sprouting. Here, we quantify bacterial and fungal contamination of seeds of 14 plant cultivars sold for home sprout production and test a range of chemical and physical methods for seed disinfestation appropriate for home use. Most seeds are contaminated with a variety of bacteria and fungi, and those microbes are usually limited to the seed surface. Heat treatments are not effective for seed disinfection because the high temperatures needed to effectively reduce microbial contamination also reduce seed germination. Two chlorine-based chemical disinfectants—dilute household bleach (0.6% sodium hypochlorite) and freshly generated hypochlorous acid (800 ppm chlorine)—were the most effective disinfection agents tested (up to a 5-log reduction in bacteria) that also did not harm seed germination.

## 1. Introduction

Germinating plant seedlings are rich in amino acids, proteins, sugars, fatty acids, and a variety of phenolics, glucosinolates, and flavonoids [1,2,3]. When grown as sprouts for human consumption, they provide a nutritious food source that can also be rich in beneficial antioxidants and bioactive compounds. However, the warm, moist conditions needed to grow nutrient-rich food sprouts simultaneously create an ideal habitat for microbial growth. While most bacterial and fungal colonists of sprouts are benign, some have significant adverse impacts if they reduce shelf life [4], affect taste or odor [5], or cause food-borne illness [6]. Each of these microbial impacts has compelled significant research and governmental regulations aimed toward minimizing microbial growth in sprouts produced commercially for human consumption [6,7,8]. Here, we examine the sources and dynamics of microbial colonization of homegrown sprouted seeds and evaluate the effectiveness of several approaches to reduce risks of unwanted microbial growth.

Some fungi and bacteria can colonize the internal tissues of a germinating seedling as endophytic symbionts [9], but many more grow on the root surface, consuming the carbon- and nitrogen-rich chemicals that rapidly growing plant roots normally exude [10]. These external rhizosphere colonists include a broad diversity of bacteria and fungi [11,12]; some have close affinities to plants as hosts, while others grow casually in the rhizosphere as saprotrophic commensals taking advantage of abundant resources. Sprouts grown for food inevitably develop a rich community of rhizosphere microorganisms; this can become problematic when the microbiome includes human pathogens such as *Salmonella* spp., toxin-producing *Escherichia coli* or *Aspergillus* spp., or plant pathogenic microbes that damage the sprouts. 

Undesirable bacteria and fungi can enter the sprouting system on or in dried plant seeds, through unhygienic conditions during sprout production, or through the post-production supply chain [6,13,14]. Contaminated seeds, however, are the most common source of sprouts-associated microbial outbreaks [15]. Because the several days of warm, moist conditions required for seeds to produce edible sprouts are also ideal for the rapid growth of microorganisms, even limited contamination of seeds can lead to unsuitably large populations of undesirable microbes in finished sprouts. For that reason, the US Food and Drug Association [7,16] and the European Sprouted Seeds Association [17] have provided guidance for the industry in the best practices for producing seeds using phytosanitary methods that ensure minimal microbial contamination, followed by disinfecting of seeds prior to use in sprout production, and microbial testing of finished sprouts before distribution [7,17]. 

There have been many efforts to develop reliable methods to reduce the microbial load that develops on sprouts grown for human consumption, including chemical disinfestation, gamma irradiation, heat, ultraviolet, and sonication [2,4,18,19,20]. Effective microbial control practices must reliably reduce microbial populations without harming seed germination or sprout growth and quality. There has been no consensus, however, on the most appropriate methods for seed disinfestation for commercial sprout production; the US and European guidance simply calls for using scientifically valid methods to reduce microbial populations. Ineffective communications about risks and appropriate mitigation measures have meant that advances in understanding how to reduce microbial growth in sprout production have not been effective at preventing outbreaks [21]. 

Homegrown sprout production avoids the risk of widespread pathogen outbreaks associated with commercial distribution of finished sprouts, but home sprout production is still vulnerable to microbial contamination. Indeed, commercial seed lots have been associated with multiple separate outbreaks [16]. However, there has been much less focus on microbial safety practices for home sprout production [22,23], and some of the more effective decontamination approaches (e.g., irradiation, ultrasound, and some chemical approaches) are not appropriate for home use [2]. With the growing popularity of the homegrown production of sprouts [24], there is a need for the evaluation of appropriate home-scale interventions to reduce microbial growth in sprout production. 

Here we present the results of a series of experiments to quantify the abundance and location of seed-borne bacteria and fungi on seeds used for homegrown sprouted seeds and test the effectiveness of a range of disinfectant agents suitable to reduce microbial growth during home sprout production. For several reasons, we chose to study the entire culturable range of bacteria and fungi rather than adopt the customary focus on the few human pathogenic bacteria commonly associated with broad outbreaks. First, the diversity of microbes that negatively affect sprout quality, safety, and nutrition is broader than those prominent pathogens. Second, testing requirements mean that seeds sold commercially for sprouting are only rarely contaminated with human pathogenic or toxin-producing microbes. We embrace the diversity of bacteria and fungi found on retail seeds as surrogates for the kinds of microbial contaminants that can enter the sprout production system through contaminated seeds and as a platform for testing disinfection processes appropriate for home production systems. 

Specifically, we quantify bacteria and fungi found on 14 cultivars of retail seeds sold for home sprouting and determine whether the microbes are on the surface or internal to the seed coats. We then conduct three overlapping experiments to evaluate the efficacy of a range of chemical and physical methods appropriate for the home disinfection of seeds as an approach to minimizing microbial growth during home sprout production.

## 2. Materials and Methods

### 2.1. Seed Sources and Traits 

Seeds of 14 plant cultivars (13 species from 5 families) were purchased commercially from True Leaf Market^TM^ (Salt Lake City, UT, USA; www.trueleafmarket.com, accessed on 22 September 2021) in sealed bags (Table 1). Seeds were handled aseptically throughout to avoid microbial contamination during laboratory processing. Seeds were certified organic (Handy Pantry^TM^ brand; Salt Lake City, UT, USA), except onion and hard red spring wheat, which were conventionally produced. 

We measured the average seed mass by weighing 100 seeds of each species (Appendix A, Table A1). We then measured the geometric traits of those 100 seeds by creating a 1600 dpi scan of the 100 seeds using an Epson Expression 1600^TM^ (Los Alamitos, CA, USA) flatbed scanner, taking care that the seeds did not touch. We used ImageJ (v 1.53 k, http://imagej.nih.gov, accessed on 1 September 2021) to measure the area, length, and width of each of the 100 seeds (Appendix A, Table A1). 

To measure seed volume, we first created standard volumetric curves for a 2-mL microcentrifuge tube (Thermo 3463; Waltham, MA, USA) and a 15-mL centrifuge tube (Falcon; Corning, NY, USA) in 10-µL or 100-µL increments with water, measuring the distance from the base of the tube to the base of the meniscus with digital calipers. We then placed the 100 seeds into the appropriate size tube and added water (measured to 1 µL) to the tubes until the seeds were completely covered by water. Tubes were briefly spun in a tabletop centrifuge to remove any trapped air bubbles. The total volume of seeds plus water was then determined using the standard volumetric curve for the tube, and the volume of 100 seeds was determined as the difference between that volume and the amount of water added. The average volume of each seed was then calculated by dividing by the number of seeds. Estimates of seed volume were performed in triplicate (Appendix A, Table A1).

### 2.2. Microbiological Media

Fungi were isolated from seeds and grown on malt extract agar (MEA) with chloramphenicol to inhibit bacterial growth (20 g malt extract, 20 g agar, 0.2 g chloramphenicol, 1 L deionized water; autoclaved together). Bacteria were isolated from seeds on one of two media: 10% trypticase soy agar (0.1TSA) with cycloheximide to inhibit fungal growth (3 g trypticase soy broth, 15 g agar, 1 L deionized water; after autoclaving, add 20 mL of a stock solution of cycloheximide (1 g /50 mL 90% ethanol) for a final concentration of 200 mg/L cycloheximide) or King’s Medium B (KB; 20 g proteose peptone #3, 1.5 g K_2_HPO_4_, 1.5 g MgSO_4_•7H_2_O, 10 mL glycerol, 15 g agar, 1 L deionized water; autoclave together).

### 2.3. Quantification of Surface and Internal Microbes on Seeds

For each seed species, we quantified the number of fungal and bacterial propagules (colony forming units; CFU) on the surface of seeds and internal to the seed coat. 

To quantify surface microbes, we aseptically placed 10 seeds in a sterile 2-mL microcentrifuge tube or (for large-seeded peas, adzuki beans, and mung beans) a 15-mL Falcon centrifuge tube (Corning 352095; Corning, NY, USA). Each tube was then filled with either 1 mL or 10 mL (for larger tubes) of sterile deionized water containing 0.01% Tween 20 as a surfactant. The tube was vortexed for 3 min to shake surface microbes into solution and then suspended in a water bath sonicator (Branson Ultrasonics™ CPX Series Ultrasonic Cleaning Bath; Emerson Electric, St. Louis, MO, USA) for an additional 1 min to further disperse microbial clusters. We then prepared three sequential 10-fold dilutions in sterile deionized water of this suspension, vortexing each dilution for 10 sec. From each dilution, 100 µL of suspension was transferred onto a 100-mm Petri dish containing either MEA or KB and spread evenly across the plate with a sterile bent glass rod. Plates were incubated at room temperature (range 22.2 to 24.6 °C) and light, and colonies were counted after 3 days for bacteria and 7 days for fungi. The dilution with the largest number of readily distinguishable colonies was used to calculate the density of microbes on a per-seed basis. Pure-culture isolates of morphologically distinguishable colonies were prepared and stored frozen in 50% glycerol at −80 °C. Quantification was replicated in two separate trials. 

To quantify microorganisms present inside the seeds, we transferred the 10 seeds that had been used for surface microbe quantification into a mesh ball tea strainer. Seeds were surface sterilized by submerging the strainer into 70% ethanol for 2 min (with agitation) followed by a 2-min soak (with agitation) in 0.6% sodium hypochlorite solution (prepared as a 10% solution of household Clorox^TM^ bleach; Oakland, CA, USA). Seeds were then rinsed twice with sterile deionized water and placed on autoclave-sterilized paper towels to pat dry. Using sterile forceps, the seeds were then transferred to pre-autoclaved 2-mL microcentrifuge tubes containing 10 2.3-mm stainless steel bearings. Each tube was filled with 2 mL 0.01% sterile Tween 20 buffer and allowed to soak for 30 min (120 min for the large seeds). Tube caps were carefully wrapped with parafilm, and then tubes were pulverized for 4 min on a Mini BeadBeater 24^TM^ (BioSpec Products; Bartlesville, OK, USA), pausing after each minute to check on tube integrity. A sterile metal probe was used to ensure all the pulverized material in the tube was in suspension, and then tubes were shaken on a vortex mixer for 30 s and then suspended in a water bath sonicator for 1 min to disperse microbial clusters. Tubes were briefly vortexed for 10 s, followed by a brief (~5 s) spin in a microcentrifuge to sediment beads and large debris. We then prepared three sequential 10-fold dilutions of the liquid suspension in sterile deionized water, vortexing each dilution for 10 s. Each dilution was transferred to MEA or KB for microbial quantification as described for surface microbes. For large-seeded species, seeds were distributed among several tubes for pulverization and then recombined prior to preparing dilutions. 

### 2.4. Initial Screening for Fungal Disinfestation and Seed Germination

We performed initial screening of a range of methods that have been suggested in the popular media for home seed disinfestation. The goal was to identify candidate disinfestation methods that significantly reduced fungal contamination of seeds prior to sprouting and that did not adversely affect germination rates. We performed these initial tests on onion seeds because our initial assessments suggested they had consistently high surface contamination with both fungi and bacteria. Seeds were aseptically removed from the source bag, and 0.25 g (58 ± 6 seeds) were placed into sterile 2-mL Eppendorf^TM^ (Hamburg, Germany) microcentrifuge tubes for each of three replicates of 25 different disinfection treatments. Chemical treatments included (1) sterile deionized water (2 min), (2) sodium hypochlorite (0.6% NaOCl; 10% household Clorox bleach, 2 min), (3) hypochlorous acid (HClO; freshly prepared Force of Nature^TM^ Multi-purpose cleaner (Westford, MA, USA), ~800 ppm chlorine, 2 min), (4) hydrogen peroxide (3%, 2 min), (5) ethanol (70%, 2 min), and (6) glacial acetic acid (5%; similar to vinegar). Each of these six chemical treatments was performed both as a simple soak with agitation and with a 2-min suspension in a water-bath sonicator. Heat treatments were conducted as (1) boiling in sterile deionized water (1 min), (2) held at 55 °C (5, 10, and 30 min), and (3) held at 70 °C (5, 10, and 30 min). Seeds were treated (50 seeds per treatment) and then placed aseptically in 1.5% water agar in a Petri dish. We recorded the percentage of seeds from which fungi were growing after 3 days and the percentage of seeds that had germination after 7 days. The seed of origin of fungal growth could not be reliably determined after 3 days, and percent germination did not meaningfully increase after 7 days. 

We compared the mean outcomes of each treatment to that of the control treatment (sterile deionized water) using a Dunnett test (DescTools package in R). A Wilcoxon paired rank test (wilcox.text, stats package in R) was used to test whether sonication significantly affected the effectiveness of the disinfectant treatments (boiling was excluded from this analysis because there was no sonication for that treatment). 

### 2.5. Factorial Evaluation of Treatments for Bacterial Disinfestation and Seed Germination

We evaluated the effectiveness of three chemical seed sterilants for disinfecting bacterial contaminants on broccoli seeds, a species we found commonly contaminated with both fungi and bacteria. Treatments included a control (sterile deionized water) and four agents: household Clorox bleach (bleach; 6% sodium hypochlorite), acetic acid (vinegar; commercial Heinz white vinegar, 5% acetic acid), hydrogen peroxide (H_2_O_2_, food grade, 30%), and hypochlorous acid (abbreviated as HCA; freshly produced Force of Nature^TM^ Multi-purpose cleaner). Although ethanol had shown promise as a disinfectant in earlier trials, it was not included here because of the difficulty of obtaining food-grade 70% ethanol for home use. Chemical sterilants were tested at a range of concentrations: Bleach (1, 2, 5, and 10 % bleach, equivalent of 0.06, 0.12, 0.30, and 0.6% sodium hypochlorite); vinegar (1, 3, and 5% acetic acid); H_2_O_2_ (3, 6, 12, and 30%); and HCA (10, 50, 100% of freshly prepared HCA, approximately 80, 400, and 800 ppm chlorine). Each of the treatments was applied for 1, 5, 10, and 15 min for full factorial treatments of concentrations (plus a sterile water control) and time of application. Each of the factorial experiments included one replicate for each sterilant concentration x time treatment combination (e.g., 5 bleach concentrations (0, 1, 2, 5, 10%) × 4 times (1, 5, 10, 15 min) = 20 treatments with 1 replicate each). Each of the full factorial experiments was conducted independently 4 times for each chemical sterilant. 

Each experimental replicate included placing 200 broccoli seeds into a clean test tube closed with a slip-on Ki-max^TM^ (Vineland, NJ, USA) cap that permits air exchange. The disinfectant solution was added to the tube to cover the seeds and allowed to soak for the designated time. The sterilant solution was decanted from the tube, and then the seeds were rinsed three times with sterile deionized water. Sterile water was added to the tube, and seeds were allowed to soak for 4 h. Water was then decanted from the tube, and seeds were kept overnight at ambient laboratory temperature and light (about 12 h dark). The next morning (~24 h after initial treatments), 9 mL of sterile deionized water was added to the tube. The tube was then vortexed for 10 s, and an aliquot of the suspension was diluted 100-fold with sterile water. This dilution was vortexed, and then an aliquot was diluted 100-fold. Aliquots (100 µL) of each of the three dilutions were individually transferred to 100-mm Petri dishes containing 0.1TSA and spread evenly with a sterile bent glass rod. This created three 100-fold dilutions, 10^−2^, 10^−4^, and 10^−6^, that allowed quantifying the number of bacterial CFU per 200 seeds with a countable range from 100 to about 10^9^ CFU per 200 seeds. The number of bacterial colonies developing on each plate was counted after 3 days of incubation at ambient laboratory conditions. The dilution with the largest number of readily distinguishable colonies was used to calculate the density of bacteria. Bacterial density was expressed as log_10_(CFU per 200 seeds + 10); the 10 was added to allow for the inclusion of replicates that recovered zero bacteria at the 10^−2^ dilution and was chosen as 10-fold below the expected minimum level of detection). Seeds from each tube were placed into a Petri dish lined with sterile filter paper; percent germination was recorded 5 days after initial treatments.

Differences among treatment means were tested using analysis of variance (function aov in R). Post-hoc comparison of means was performed using Tukey’s HSD multiple comparisons test (TukeyHSD in R) with 95% confidence. 

### 2.6. Direct Comparison of Disinfection Agents on Bacteria, Fungi, and Seed Germination

We brought together insights from the initial screening and factorial experiments for a final test of the effects of six disinfection treatments on the bacterial and fungal loads on seedlings after 24 h and on seed germination success. Disinfection treatments were bleach (10% commercial Clorox^TM^, 0.6% sodium hypochlorite), hypochlorous acid (HCA; freshly produced Force of Nature^TM^ Multi-purpose cleaner, ~800 ppm chlorine), vinegar (diluted Heinz^TM^ (Sharpsburg, PA, USA) white vinegar, 3% acetic acid), H_2_O_2_ (3%), 55 °C water; Sonication in 23 °C (room temperature) water, and a control treatment of sterile deionized water (23 °C). Note that H_2_O_2_ was used at 3% rather than the higher concentrations tested earlier because concentrations of food-grade H_2_O_2_ at higher concentrations are widely restricted for consumer purchase. Each treatment was replicated 5 times. The entire experiment was conducted twice with treatments lasting 5 min and twice for treatments lasting 15 min. 

To conduct each experiment, 200 broccoli seeds were placed in sterile test tubes with a slip-on cap (35 tubes total). The disinfecting agent was poured into the tubes to cover the seeds. At the conclusion of the treatment time, the agent was decanted from the tubes, and seeds were rinsed 3 times with sterile deionized water. Sterile deionized water was then added to each tube to cover seeds and allowed to soak for 4 h before draining. At 24 h from the initial treatment, 9 mL of sterile deionized water was added to each tube. Tubes were vortexed for 10 s, then two 100-fold dilutions of the solution were prepared. This created three 100-fold dilutions: 10^−2^, 10^−4^, and 10^−6^; aliquots (100 µL) of each of the three dilutions were individually transferred to 100-mm Petri dishes containing 0.1TSA and spread evenly with a sterile bent glass rod. The same was done for the 10^−2^ on MEA medium for quantification of fungi. Seeds from each tube were then placed into a Petri dish lined with sterile filter paper; percent germination was recorded 5 days after initial treatments. Bacterial and fungal densities were expressed as log_10_(CFU per 200 seeds + 10); the 10 was added to allow for the inclusion of replicates that recovered zero bacteria at the 10^−2^ dilution and was chosen as 10-fold below the expected minimum level of detection). Percent seed germination was measured after 5 days.

Differences among treatment means were tested using analysis of variance, treating each experimental repetition as a block (function aov in R). Soaking times of 5 min and 15 min were analyzed separately. Least squares means and 95% confidence intervals for the mean of each treatment were calculated using emmeans function (emmeans package in R). Post-hoc comparison of means was performed using Tukey’s HSD multiple comparisons test (TukeyHSD in R) with 95% confidence.

## 3. Results

### 3.1. Microbial Loads of Seeds for Sprouting

**Figure 1:**
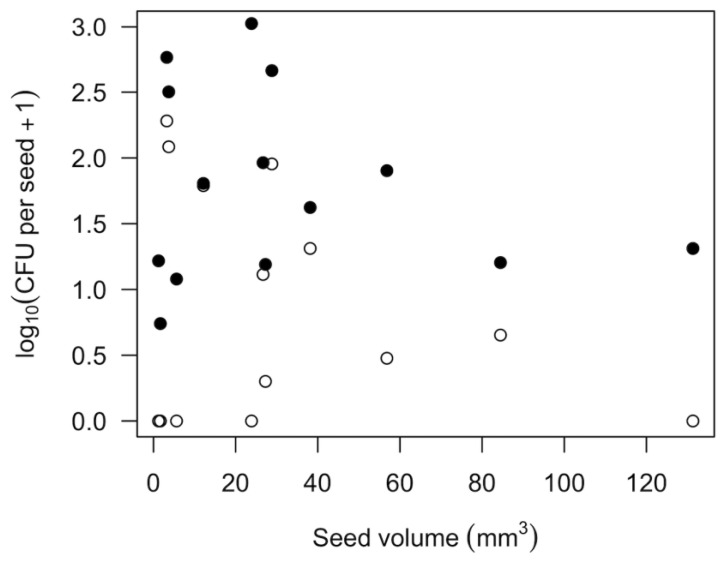
Number of fungal (open circles) or bacterial (closed circles) propagules (CFU; colony forming units) on the exterior of 14 types of seeds. Each point is the average of two replicates per species, as given in Table 2.

Bacterial and fungal propagules were commonly found on the surfaces of most types of seeds but seldom in the seed interior. Of the 14 types of seeds commercially sold for home sprouting that we tested, all were infested with bacteria and 9 with fungi (Table 2). Fungi were found almost exclusively on the surface of seeds. Bacteria were absent or very rare in the seed interior, with the exception of buckwheat groats. While the number of CFU per seed varied across replicate trials, the patterns were consistent for which species tended to have more or less microbial contamination. Legume species (Fabaceae) as a whole had lower numbers of surface microbial CFU per seed for fungi (1.1 ± s.e. 0.6, *n* = 5 spp.) and bacteria (24.7 ± 11.1) than seeds of grasses (Poaceae; fungi 30.1 ± 20.0, *n* = 4 spp.; bacteria 150.8 ± 104.4) and mustards (Brassicaceae; fungi 90.50 ± 30.00, *n* = 2 spp.; bacteria 190.0 ± 127.0). Onion seeds also had abundant fungi and bacteria (Table 2). Seed size was not a predictor of microbial CFU per seed (Figure 1; *p* > 0.3 for both fungi and bacteria). 

While characterizing the taxonomic composition of the microbiomes of seeds was outside the scope of this study, we observed a broad morphological diversity of Fungi (commonly *Mucor*, *Aspergillus*, *Cladosporium*, and *Alternaria* spp.) and Bacteria (commonly Gammaproteobacteria and Bacilli).

### 3.2. Initial Screening of Treatments for Fungal Disinfestation and Seed Germination

We conducted an initial evaluation of the effectiveness of fungal disinfectants by measuring the proportion of seeds from which fungal growth initiated and the proportion of onion seeds that germinated. Effective disinfectants must significantly reduce the percentage of seeds with fungi while not significantly reducing the percent germination. Initial screens for fungal disinfectants compared the percentage of onion seeds from which fungi grew and the proportion of seeds that germinated to the control treatment of sterile deionized water (2 min, agitated). All of the treatments significantly reduced fungal growth except for sonication, hydrogen peroxide, and 55 °C for 5 min with agitation (Table 3). All treatments at 70 °C were lethal to seeds (Table 3). There was no consistent effect of sonication on fungal growth (Table 3). The most effective treatments to reduce fungal growth on seeds while not harming germination rates were sodium hypochlorite (bleach), ethanol, and glacial acetic acid (vinegar), with hypochlorous acid also showing potential as an effective treatment. 

### 3.3. Evaluation of Treatments for Bacterial Disinfestation and Seed Germination

**Figure 2:**
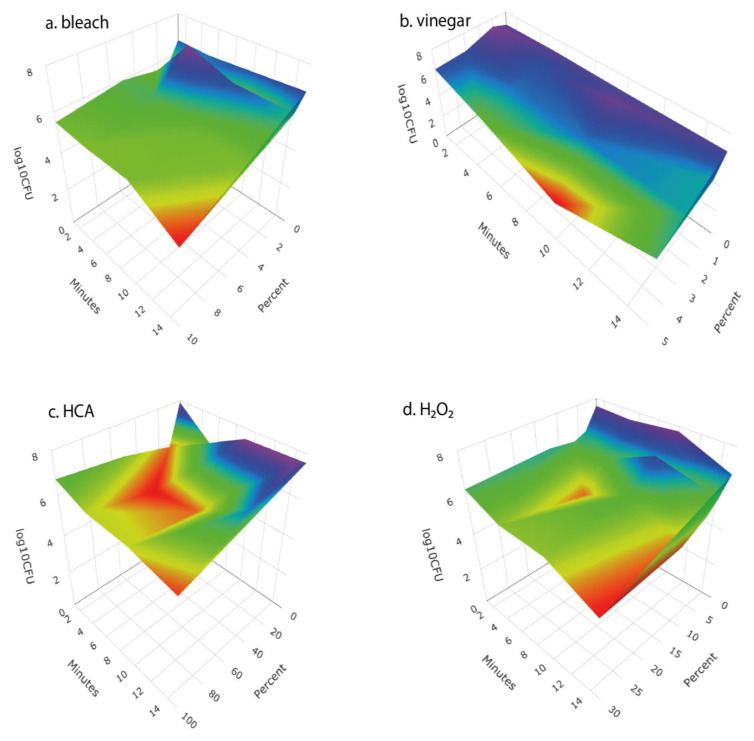
Response surface of the antibacterial effectiveness of (**a**) bleach (given as percent of household bleach), (**b**) vinegar (given as % acetic acid), (**c**) hypochlorous acid (given as percent of Force of Nature cleaner), and (**d**) hydrogen peroxide (as percent) at different concentrations and times of treatment. Surface represents a fit to the means of four replicates for concentration × time combination.

**Figure 3:**
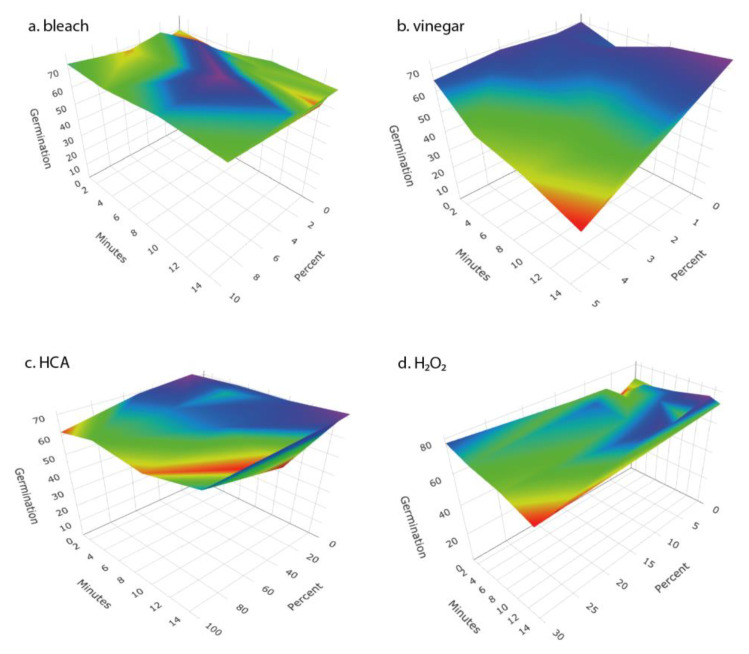
Response surface of the impact on germination of broccoli seeds of (**a**) bleach (given as percent of household bleach), (**b**) vinegar (given as % acetic acid), (**c**) hypochlorous acid (given as percent of Force of Nature cleaner), and (**d**) hydrogen peroxide (as percent) at different concentrations and times of treatment. Surface represents a fit to the means of four replicates for concentration × time combination.

We examined the response surfaces of antibacterial effectiveness and the impact on seed germination for a range of concentrations of four disinfectant solutions over time periods from 1 to 15 min. For each of the disinfectants, antibacterial effectiveness increased with disinfectant concentration and length of soaking (Figure 2). Vinegar, HCA, and H_2_O_2_ each caused meaningful reductions in seed germination, whereas bleach did not (Figure 3). 

### 3.4. Testing of Optimal Disinfectant Treatments for Bacterial Disinfestation and Seed Germination

**Figure 4:**
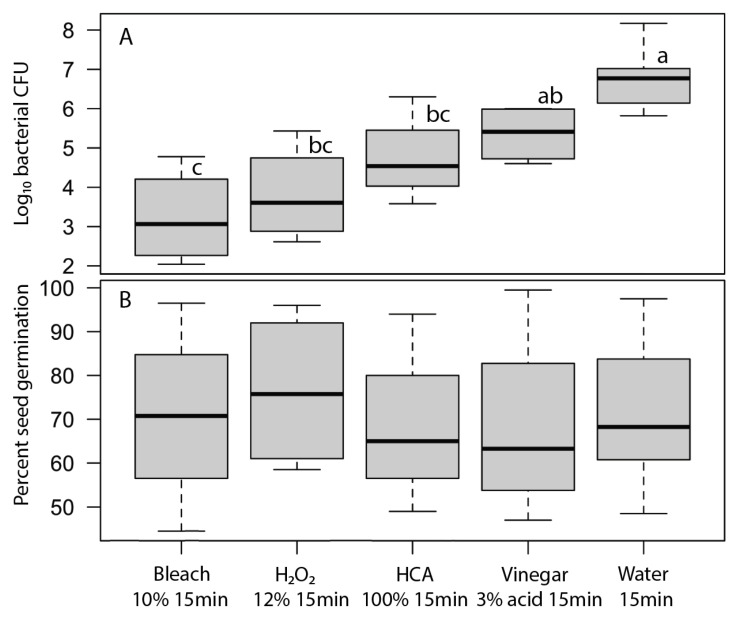
Most effective concentration and treatment times for each of the bacterial disinfectant agent treatments; (**A**) bacterial density on broccoli sprouts after 24 h and (**B**) germination after 7 days. Mean bacterial densities (log10 CFU per 200 seeds) were significantly different among treatments (F_4,27_ = 19.3, *p* ≤ 0.00001); treatments with the same letter were not significantly different (Tukey’s HSD test, 95% confidence interval). Mean germination rates were not significantly different among treatments (F_4,27_ = 0.15, *p* = 0.96). Boxplots show the median as a thick line, boxes are delimited by 1st and 3rd quartiles, and whiskers show the range of observed values.

For each disinfectant agent, we selected the concentration x time treatment that produced the greatest reduction in bacteria while not causing a significant loss of broccoli seed germination by examining plots of log_10_ bacterial CFU vs. seed germination. Those optimal treatments for each agent were 10% bleach for 15 min, 3% vinegar (i.e., 3% acid) for 15 min, 100% HCA for 15 min, 12% H_2_O_2_ for 15 min (Figure 4). The bleach treatment provided more than a 3-log reduction in bacteria that developed on the sprouting seeds after 24 h. The bacterial reductions by H_2_O_2_ and HCA were numerically more modest but were not statistically significantly different from the bleach treatment. Vinegar was not effective in reducing bacteria when used at concentrations that did not also impede seed germination. 

### 3.5. Direct Comparison of Disinfection Agents on Bacteria, Fungi, and Seed Germination

**Figure 5:**
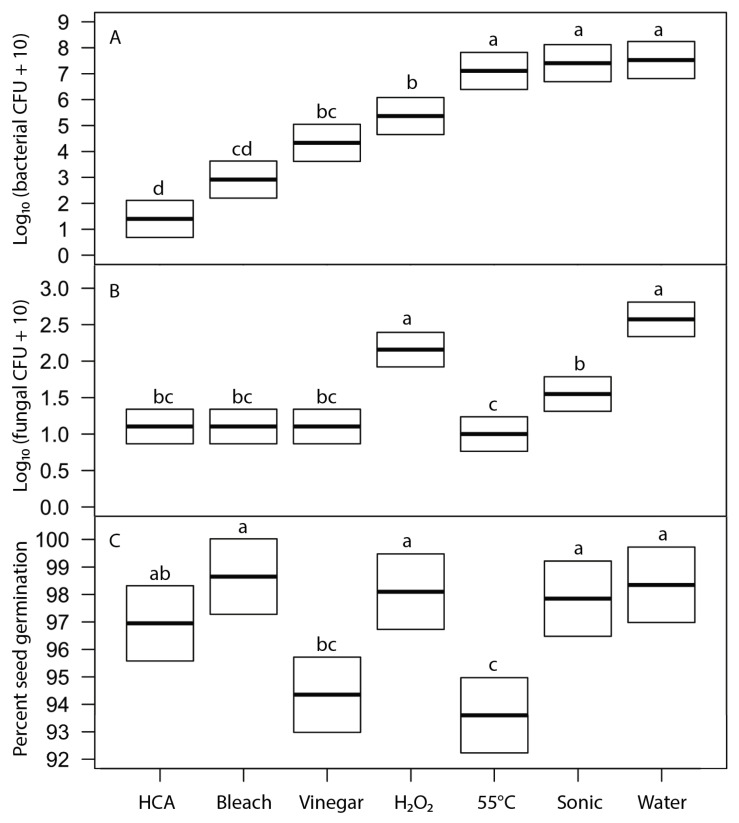
Effects of 15-min treatment with disinfection agents on (**A**) bacterial load on 24-h broccoli sprouts (F6,61 = 7.5, *p* ≤ 5 × 10^−6^), (**B**) fungal load on 24-h sprouts (F6,61 = 7.5, *p* ≤ 2 × 10^−16^), and (**C**) broccoli seed germination after 5 days (F6,61 = 7.5, *p* ≤ 0.0044). Thick line indicates least-square mean of treatment; box shows the 95% confidence intervals for the estimate of the mean. Within each frame, means of treatments with the same letter were not significantly different (Tukey’s HSD test, 95% confidence interval).

**Figure 6:**
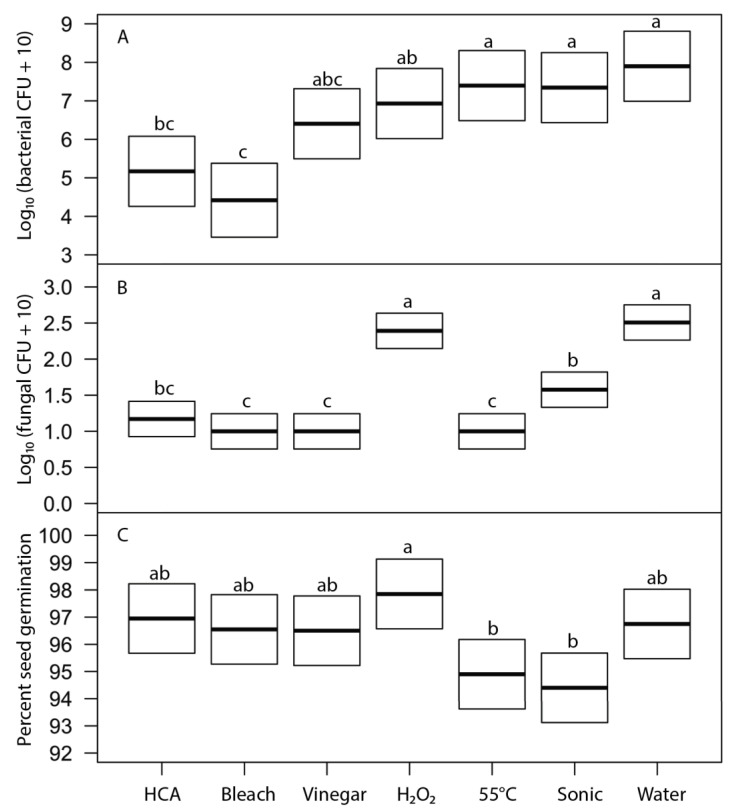
Effects of 5-min treatment with disinfection agents on (**A**) bacterial load on 24-h broccoli sprouts (F_6,61_ = 7.5, *p* ≤ 2 × 10^−16^), (**B**) fungal load on 24-h sprouts (F_6,61_ = 7.5, *p* ≤ 1.1 × 10^−15^), and (**C**) broccoli seed germination after 5 days (F_6,61_ = 7.5, *p* ≤ 5.8 × 10^−7^). Thick line indicates least-square mean of treatment; box shows the 95% confidence intervals for the estimate of the mean. Within each frame, means of treatments with the same letter were not significantly different (Tukey’s HSD test, 95% confidence interval).

We evaluated the effects of six disinfection agents on bacterial and fungal growth on sprouts and on seed germination using broccoli seeds. These agents represent treatments suggested for home use in popular literature. Soaking for 15 min in freshly prepared hypochlorous acid (approximately 800 ppi chlorine) and sodium hypochlorite (0.6%, prepared as diluted household bleach) were the most effective treatments at reducing bacterial growth (5 to 4 log reductions) and fungal growth (to undetectable levels) on sprouts, without significantly reducing seed germination (Figure 5). Vinegar soak was moderately effective as a disinfectant but significantly reduced seed germination. Hydrogen peroxide, heat treatment at 55 °C, and sonication were not effective treatments. Because 15-min soak periods are longer than is often acceptable for home production, we also tested all the same agents with a 5-min soak (Figure 6). Observed patterns were similar to those at 15 min but much less effective at reducing bacterial growth (only 2 to 3 log reductions for HCA and bleach treatments). 

## 4. Discussion

All seeds are routinely contaminated with a diversity of microbes. The majority of those microbes are benign, neither human nor plant pathogens. The likelihood of encountering human pathogenic bacteria on seeds sold for home sprouting is reduced even further because best practices are designed specifically to avoid contamination with those bacteria [7,16,17]. Such practices do not avoid general microbial contamination, however, and contaminated seed is still the most common source of sprout-associated human pathogen outbreaks [15]. Because the warm temperatures and continuous moisture required to produce sprouts for human consumption are the same as those for the robust growth of fungi and bacteria, minimizing the number of microbial propagules present at the start of home seed sprouting is important to reduce the risk of harmful microbial growth during the sprouting process. Harmful microbial growth can include biofilms that affect sprout taste, appearance, and shelf life, as well as human pathogens. Safe, effective, and convenient seed disinfection protocols are needed for home sprout production. 

Fungi and bacteria can colonize both the interior and the surface of the seeds. In our survey of 14 types of seeds sold for home sprouting, we found that internal colonization was extremely rare (Table 3). This is important for sprout production because it is much easier to disinfect the seed surface than the inside of seeds. The quantity of bacteria and fungi varied greatly across seeds of different plant species, and this variation was not predictably related to seed size (Table 1, Figure 1). Instead, variation in microbial density is likely associated with seed surface roughness [25] and the seed production environment. A broad survey of the abundance and taxonomic composition of surface microbiomes of diverse seed sources and plant species would help provide a clearer picture of which kinds of microbes are most likely to present challenges for the production of different kinds of home sprouts. 

Seed surfaces can be disinfected using either physical (heat, washing, or sonication) or chemical agents. The key challenge in seed disinfection for home sprouting is to provide a safe and convenient approach that is effective at reducing microbial loads while not adversely affecting seed germination rates. In our tests, heat treatments that were stringent enough to meaningfully reduce microbial contamination also significantly reduced seed germination (Table 3, Figure 5 and Figure 6). While it may be possible to fine-tune the temperature and duration of heat treatments to reduce microbial loads without harming germination for particular microbe-plant species combinations, it is unlikely that a single heat-treatment protocol would be broadly effective for broad inclusion in home sprout production. 

Bath sonication is often used in microbiology to dislodge and disperse biofilms and clumps of microorganisms to facilitate the quantification of microbes using cultural methods [26]. Suitable ultrasonic bath cleaners are safe, inexpensive, and readily available for home jewelry cleaning, so we tested sonication as a potential physical means to dislodge microbes from seeds so that propagules could then be removed by simple rinsing. We found that sonication did reduce fungal propagules without affecting seed germination, but it had no effect on bacterial load (Table 3, Figure 5 and Figure 6). Sonication does not seem promising for home seed disinfection.

We tested a number of chemical treatments that are commonly used as disinfectants, including ethanol, hydrogen peroxide, vinegar (acetic acid), household bleach (sodium hypochlorite), and hypochlorous acid. Ethanol (70%, laboratory-grade) was found to be an effective disinfectant without harming seed germination (Table 3) but was discontinued from further testing because food-grade ethanol at 70% concentration is difficult to obtain for home use; ethyl alcohol sold for antiseptic use is denatured and contains chemical additives that are toxic if ingested and has a strong, unpleasant odor. Similarly, high concentrations of food-grade hydrogen peroxide (30% or 12% H_2_O_2_) showed strong antimicrobial effectiveness (Figure 2 and Figure 4) with only minimal impact on seed germination (Figure 3) but concentrated H_2_O_2_ poses hazards as a corrosive agent, strong oxidizer, and potential for explosion, and so is not suitable for safe and effective home use as a seed disinfectant. The lower, safer concentration of readily available 3% H_2_O_2_ was not consistently effective at reducing microbial loads (Figure 5 and Figure 6). 

Household vinegar is a solution of 5% acetic acid, and it is food-safe and readily available in most kitchens. At that concentration, vinegar is an effective seed disinfectant (Table 3 and Figure 2). Unfortunately, it can also strongly reduce seed germination (Table 3 and Figure 3). Diluted vinegar (to 3% acetic acid) was only moderately effective at reducing the growth of fungi and bacteria (Figure 4, Figure 5 and Figure 6) and sometimes significantly reduced germination rates (Figure 5), probably due to the phytotoxic effects of acetic acid [27]. Similar to heat treatments, it might be possible to find optimal concentrations and soak times of vinegar that limit microbial growth while not adversely affecting the germination of particular seed species, but our data suggest that vinegar is unlikely to be effective for general use in home seed disinfestation. 

The two most effective seed disinfectant solutions were chlorine based: dilute household bleach (NaOCl; 0.6% sodium hypochlorite) and freshly prepared hypochlorous acid (HClO; 800 ppm chlorine). Both consistently reduced fungal growth on germinating seeds (by up to a 5-log unit reduction in bacteria) without having a significant effect on seed germination (Table 3 and Figure 2, Figure 3, Figure 4, Figure 5 and Figure 6). Sodium hypochlorite has a long history as an effective disinfectant for seeds [28,29]. It is widely available, inexpensive, and breaks down quickly in light, but it is also caustic due to its high alkalinity (pH 11 or more). Additionally, some growers concerned with using organic methods for food production may be hesitant to use bleach; it is allowable in USDA-certified organic production only if residual chlorine levels do not exceed 4 ppm, similar to what is in domestic water sources and much lower than concentrations used to sterilize seeds (USDA Organic Regulations 7 CFR § 205.601 and § 205.605). 

In contrast to sodium hypochlorite, hypochlorous acid is a weak acid (HClO, pH 6.8) that is a powerful oxidizing agent with disinfectant properties. It is regarded as non-hazardous by the US Environmental Protection Agency, widely used in the food service industry for disinfection of both surfaces and fresh-cut vegetables, has less of an odor than bleach, and is relatively non-corrosive [30,31]. Hypochlorous acid is less widely used than bleach because it must be prepared fresh by electrolysis of a weak solution of table salt (NaCl); hypochlorous acid is sometimes referred to as ‘slightly acidic electrolyzed water’. Fresh production is needed because hypochlorous acid is unstable, reverting back to the salt solution and losing its antimicrobial effectiveness over the course of a few days [32]. A number of simple and inexpensive kits to produce fresh hypochlorous acid at home from tap water and table salt are commercially available. In our study, hypochlorous acid was consistently as effective as sodium hypochlorite and was easier to handle because of the lack of corrosiveness. Hypochlorous acid is not, however, currently allowed in USDA certified organic production (allowed chlorine materials include only calcium hypochlorite, chlorine dioxide, sodium hypochlorite, and acidified sodium chlorite per USDA Organic Regulations 7 CFR § 205.601 and § 205.605). While effective and easy to use, its short shelf life, the need to produce fresh solution through electrolysis, and lack of organic certification may inhibit widespread adoption for home sprout production.

## 5. Conclusions

Minimizing growth of bacterial and fungal growth during the home growth of sprouted seeds helps produce a safe, healthy, sustainable, and nutritious food source. Because an important source of microbial contamination in sprout production is from bacterial and fungal propagules on the surface of seeds, effective surface disinfection of seeds is critical to reducing microbial load on sprouts. Heat treatments that significantly reduce microbial growth also reduce seed germination and so are not likely to be effective approaches to seed disinfection. Of several widely recommended chemical disinfectants, chlorine-based sodium hypochlorite (dilute household bleach) and hypochlorous acid (slightly acidic electrolyzed water) were the most effective, reducing bacterial loads by 5-log units or more without negatively affecting seed germination.

## Figures and Tables

**Table 1:** Species of plant seeds used for assessment of seed-borne microbial contamination.

Family	Species	Scientific Name
Amaryllidaceae	Onion	*Allium cepa*
Brassicaceae	Broccoli	*Brassica oleracea* var. *italica*
Brassicaceae	Radish, China Rose	*Raphanus sativus*
Fabaceae	Lentils	*Lens culinaris*
Fabaceae	Alfalfa	*Medicago sativa*
Fabaceae	Peas	*Pisum sativum*
Fabaceae	Clover	*Trifolium pratense*
Fabaceae	Adzuki bean	*Vigna angularis*
Fabaceae	Mung bean	*Vigna radiata*
Poaceae	Barley	*Hordeum vulgare*
Poaceae	Millet	*Panicum miliaceum*
Poaceae	Wheat, hard red spring	*Triticum aestivum*
Poaceae	Wheat, hard white	*Triticum aestivum*
Polygonaceae	Buckwheat groats	*Fagopyrum esculentum*

**Table 2:** Colony-forming units (CFU) per plant seed of fungi (on MEA medium) or bacteria (on KB medium) from dilution plating of microbes from seed surface or from interior of surface-sterilized seeds.

	Seed Surface				Seed Interior			
Species	Fungi		Bacteria		Fungi		Bacteria	
	Rep1	Rep2	Rep1	Rep2	Rep1	Rep2	Rep1	Rep2
Onion	160	220	640	520	0	0	5	3
Broccoli	240	1	610	24	0	0	0	0
Radish, China Rose	120	1	100	26	0	0	0	0
Lentils	2	0	13	16	0	0	0	0
Alfalfa	0	0	11	20	0	0	0	0
Peas	0	0	23	16	0	0	1	5
Clover	0	0	1	8	0	0	2	0
Adzuki bean	6	1	22	8	0	0	0	1
Mung bean	1	3	64	94	0	0	0	0
Barley	157	21	500	420	0	0	2	0
Millet	0	0	21	1	0	1	0	0
Wheat, hard red spring	20	4	162	20	0	0	0	0
Wheat, hard white	35	4	65	17	0	0	0	0
Buckwheat Groats	0	0	2000	120	0	0	750	2

**Table 3:** Effect of chemical and heat treatments on fungal growth and germination of onion seeds (*n* = 3 per treatment). Treatments that significantly reduced the percentage of seeds with fungal growth compared to the water control are indicated with an * (Dunnett’s test, alpha = 0.01; F_24,50_ = 31.9, *p* ≤ 0.0001) and those that significantly reduced seed germination are indicated with a ^#^ (Dunnett’s test, alpha = 0.01; F_24,50_ = 21.9, *p* ≤ 0.0001). Means are compared to the control treatment of sterile deionized water (2 min, agitated). Paired comparisons showed no consistent effect of sonication on fungal growth (Wilcoxon V = 37, *p* = 0.36) or seed germination (V = 37, *p* = 0.76).

	Fungus (% ± sd)		Germination (% ± sd)	
Treatment	Agitated	Sonicated	Agitated	Sonicated
Sterile deionized water (2 min)	98.9 ± 1.9	75.8 ± 12.4	79.7 ± 4.4	68.5 ± 9.0
Sodium hypochlorite (0.6%, 2 min)	21.1 ± 16.5 *	15.9 ± 10.1 *	78.3 ± 2.5	71.6 ± 22.0
Ethanol (70%, 2 min)	0.0 ± 0.0 *	0.6 ± 1.0 *	81.5 ± 5.6	89.7 ± 4.3
Hypochlorous acid (2 min)	55.4 ± 6.5 *	64.7 ± 11.3 *	85.8 ± 2.7	94.7 ± 5.4
Hydrogen Peroxide (3%, 2 min)	96.1 ± 2.8	82.4 ± 7.8	80.4 ± 10.0	70.8 ± 8.8
Glacial acetic acid (5%, 2 min)	0.6 ± 1.0 *	0.6 ± 1.0 *	71.9 ± 3.8	41.3 ± 33.3 ^#^
55 °C (5 min)	80.2 ± 0.7	16.9 ± 9.3 *	80.6 ± 19.4	84.3 ± 7.6
55 °C (10 min)	37.6 ± 14.5 *	56.6 ± 34.1 *	67.2 ± 18.7	82.7 ± 11.7
55 °C (30 min)	41.8 ± 19.2 *	14.9 ± 13.6 *	56.2 ± 14.0	65.5 ± 14.3
70 °C (5 min)	1.1 ± 1.9 *	3.2 ± 5.5 *	8.0 ± 3.4 ^#^	8.0 ± 11.1 ^#^
70 °C (10 min)	1.1 ± 1.9 *	3.9 ± 5.5 *	8.4 ± 1.6 ^#^	2.8 ± 4.9 ^#^
70 °C (30 min)	0.0 ± 0.0 *	0.0 ± 0.0 *	5.3 ± 2.3 ^#^	0.0 ± 0.0 ^#^
Boiling (1 min)	0.0 ± 0.0 *		57.0 ± 6.1	

## Data Availability

The data used to support the findings of this study can be made available by the corresponding author upon request.

## Appendix A. Seed Size Traits Plant Species Used for Analysis of Microbial Contamination and Disinfestation Processes

**Table A1:** Traits of individual seeds for plant species used in this study, as given in Table 1. Values are mean ± standard deviation. Area, length, and width are based on 100 seeds. Volume is based on triplicate measurements of 100 seeds.

Family	Scientific Name	Area (mm^2^)	Length (mm)	Width (mm)	Volume (mm^3^)	Mass (g/100)
Amaryllidaceae	*Allium cepa*	5.16 ± 0.59	2.91 ± 0.19	2.26 ± 0.21	3.24 ± 0.20	0.435
Brassicaceae	*Brassica oleracea*	2.10 ± 0.45	1.79 ± 0.21	1.48 ± 0.18	3.72 ± 0.85	0.375
Brassicaceae	*Raphanus sativa*	7.69 ± 1.13	3.53 ± 0.27	2.76 ± 0.27	12.15 ± 3.28	1.328
Fabaceae	*Lens culinaris*	18.56 ± 2.36	4.98 ± 0.30	4.73 ± 0.34	27.26 ± 1.23	3.499
Fabaceae	*Medicago sativa*	2.55 ± 0.35	2.30 ± 0.22	1.41 ± 0.11	1.24 ± 0.88	0.219
Fabaceae	*Pisum sativum*	29.92 ± 3.83	6.51 ± 0.45	5.83 ± 0.43	131.31 ± 1.17	15.709
Fabaceae	*Trifolium pratense*	2.32 ± 0.39	2.06 ± 0.19	1.43 ± 0.15	1.68 ± 0.15	0.224
Fabaceae	*Vigna angularis*	25.19 ± 2.99	6.22 ± 0.44	5.14 ± 0.33	84.47 ± 3.04	11.007
Fabaceae	*Vigna radiata*	19.12 ± 2.11	5.64 ± 0.42	4.31 ± 0.23	56.82 ± 1.92	7.863
Poaceae	*Hordeum vulgare*	18.26 ± 1.99	7.14 ± 0.48	3.25 ± 0.20	28.82 ± 1.14	3.601
Poaceae	*Panicum miliaceum*	4.99 ± 0.28	2.76 ± 0.13	2.30 ± 0.10	5.62 ± 0.70	0.631
Poaceae	*Triticum aestivum* (red)	15.38 ± 2.58	6.29 ± 0.54	3.10 ± 0.36	26.68 ± 0.52	3.454
Poaceae	*T. aestivum* (white)	18.27 ± 2.01	6.58 ± 0.42	3.53 ± 0.27	38.15 ± 0.87	4.735
Polygonaceae	*Fagopyrum esculentum*	11.70 ± 1.57	4.20 ± 1.10	3.62 ± 0.41	23.87 ± 0.85	2.425

## References

[1] Marton M., Mandoki Z., Csapo-Kiss Z., Csapo J. (2010). The role of sprouts in human nutrition. A review. Acta Univ. Sapientiae.

[2] Mir S.A., Farooq S., Shah M.A., Sofi S.A., Dar B., Hamdani A.M., Khaneghah A.M. (2021). An overview of sprouts nutritional properties, pathogens and decontamination technologies. LWT.

[3] Chavan J., Kadam S., Beuchat L.R. (1989). Nutritional improvement of cereals by sprouting. Crit. Rev. Food Sci. Nutr..

[4] Rajkowski K.T., Thayer D.W. (2001). Alfalfa seed germination and yield ratio and alfalfa sprout microbial keeping quality following irradiation of seeds and sprouts. J. Food Prot..

[5] Sikin A.M., Zoellner C., Rizvi S.S. (2013). Current intervention strategies for the microbial safety of sprouts. J. Food Prot..

[6] Miyahira R.F., Antunes A.E.C. (2021). Bacteriological safety of sprouts: A brief review. Int. J. Food Microbiol..

[7] Code of Federal Regulations (2015). Section 112.142 Standards for the growing, harvesting, packing, and holding of produce for human consumption, Subpart M—Sprouts. Title 21 Food and Drugs, Subchapter B Food for Human Consumption.

[8] Ding H., Fu T.J., Smith M.A. (2013). Microbial contamination in sprouts: How effective is seed disinfection treatment?. J. Food Sci..

[9] Verma S.K., Kharwar R.N., White J.F. (2019). The role of seed-vectored endophytes in seedling development and establishment. Symbiosis.

[10] Zhalnina K., Louie K.B., Hao Z., Mansoori N., da Rocha U.N., Shi S., Cho H., Karaoz U., Loqué D., Bowen B.P. (2018). Dynamic root exudate chemistry and microbial substrate preferences drive patterns in rhizosphere microbial community assembly. Nat. Microbiol..

[11] Kim S.Y., Ban G.-H., Hong Y.W., Jang M.J., Kim S.A. (2022). Microbiome shifts in sprouts (alfalfa, radish, and rapeseed) during production from seed to sprout using 16S rRNA microbiome sequencing. Food Res. Int..

[12] Tournas V. (2005). Moulds and yeasts in fresh and minimally processed vegetables, and sprouts. Int. J. Food Microbiol..

[13] Sarno E., Pezzutto D., Rossi M., Liebana E., Rizzi V. (2021). A review of significant European foodborne outbreaks in the last decade. J. Food Prot..

[14] Escamilla D., Rosso M.L., Zhang B. (2019). Identification of fungi associated with soybeans and effective seed disinfection treatments. Food Sci. Nutr..

[15] Obenhuber D.C., Cloyd T.C., Fields A., Homola P. (2020). Memorandum of Record. 2012–2020 Sprout-Related Outbreak Data. https://www.regulations.gov/document/FDA-2014-N-0053-0096.

[16] Office of Food Safety (2022). Reducing Microbial Food Safety Hazards in the Production of Seed for Sprouting: Guidance for Industry.

[17] European Sprouted Seeds Association (2017). ESSA Hygiene Guideline for the Production of Sprouts and Seeds for Sprouting.

[18] Ngnitcho P.-F.K., Khan I., Tango C.N., Hussain M.S., Oh D.H. (2017). Inactivation of bacterial pathogens on lettuce, sprouts, and spinach using hurdle technology. Innov. Food Sci. Emerg. Technol..

[19] Kumar S., Gautam S. (2019). A combination process to ensure microbiological safety, extend storage life and reduce anti-nutritional factors in legume sprouts. Food Biosci..

[20] Zhang C., Zhang Y., Zhao Z., Liu W., Chen Y., Yang G., Xia X., Cao Y. (2019). The application of slightly acidic electrolyzed water in pea sprout production to ensure food safety, biological and nutritional quality of the sprout. Food Control..

[21] Erdozain M.S., Allen K.J., Morley K.A., Powell D.A. (2013). Failures in sprouts-related risk communication. Food Control..

[22] Harmon S.M., Kautter D.A., Solomon H.M. (1987). *Bacillus cereus* contamination of seeds and vegetable sprouts grown in a home sprouting kit. J. Food Prot..

[23] Mulaosmanovic E., Farkas S., Vågsholm I., Darlison J., Sousa M., Mogren L., Gharaie S., Alsanius B.W. (2019). Safety risks associated with dispersal of *E. coli* O157: H7 in home sprouting modules. LWT Food Sci. Technol..

[24] Ebert A.W. (2022). Sprouts and microgreens—Novel food sources for healthy diets. Plants.

[25] Fransisca L., Feng H. (2012). Effect of surface roughness on inactivation of *Escherichia coli* O157: H7 87-23 by new organic acid–surfactant combinations on alfalfa, broccoli, and radish seeds. J. Food Prot..

[26] Kobayashi H., Oethinger M., Tuohy M.J., Procop G.W., Bauer T.W. (2009). Improved detection of biofilm-formative bacteria by vortexing and sonication: A pilot study. Clin. Orthop. Relat. Res..

[27] Lynch J. (1977). Phytotoxicity of acetic acid produced in the anaerobic decomposition of wheat straw. J. Appl. Bacteriol..

[28] Abdul-Baki A.A., Moore G.M. (1979). Seed disinfection with hypochlorites: A selected literature review of hypochlorite chemistry and definition of terms. J. Seed Technol..

[29] Ganiyu S., Popoola A., Imonmion J., Uzoemeka I., Ojo K. (2021). Effect of three sterilizing agents on seed viability, seedling vigor and occurrence of seed-borne bacterial pathogens of two tomato cultivars. Niger. J. Plant Prot..

[30] Ampiaw R.E., Yaqub M., Lee W. (2021). Electrolyzed water as a disinfectant: A systematic review of factors affecting the production and efficiency of hypochlorous acid. J. Water Process Eng..

[31] Rahman S., Khan I., Oh D.H. (2016). Electrolyzed water as a novel sanitizer in the food industry: Current trends and future perspectives. Compr. Rev. Food Sci. Food Saf..

[32] Block M.S., Rowan B.G. (2020). Hypochlorous acid: A review. J. Oral Maxillofac. Surg..

